# The effect of sex on the isolated and combined α‐ and β‐adrenergic control of blood flow during handgrip in adults at high altitude: An exploratory study

**DOI:** 10.14814/phy2.70754

**Published:** 2026-02-03

**Authors:** Lauren E. Maier, Emily R. Vanden Berg, Lydia Simpson, Michiel Ewalts, Katharine Foster, Jared Baylis, Christopher Gasho, David Macleod, Sean van Diepen, James Anholm, Justin Lawley, Philip N. Ainslie, Travis D. Gibbons, Michael Stembridge, Jonathan Moore, Craig D. Steinback

**Affiliations:** ^1^ Neurovascular Health Laboratory, Faculty of Kinesiology, Sport, and Recreation University of Alberta Edmonton Alberta Canada; ^2^ Centre for Heart, Lung and Vascular Health, School of Health and Exercise Sciences University of British Columbia—Okanagan Kelowna British Columbia Canada; ^3^ University of Bristol Bristol UK; ^4^ University of Innsbruck Innsbruck Austria; ^5^ Bangor University Bangor UK; ^6^ Cardiff School of Sport and Health Sciences Cardiff Metropolitan University Cardiff UK; ^7^ Loma Linda University Loma Linda California USA; ^8^ Southern Medicine Program University of British Columbia Kelowna British Columbia Canada; ^9^ Duke University Durham North Carolina USA; ^10^ Faculty of Medicine and Dentistry University of Alberta Edmonton Alberta Canada; ^11^ Northern Arizona University Flagstaff Arizona USA

**Keywords:** exercise, handgrip, high altitude, hypoxia, sex differences

## Abstract

This study examined how sex influences blood flow during exercise at altitude and relative contributions of adrenergic mechanisms. Thirteen participants (8 M/5F) were tested at low and high altitude (days 3–11). Participants performed rhythmic handgrip for 3 min at 25% maximal voluntary contraction during local infusions of saline, propranolol (β‐adrenergic blockade), and phentolamine with propranolol (α‐β‐adrenergic blockade). Doppler ultrasound was used to examine brachial artery blood flow (FBF) and calculate forearm vascular conductance (FVC). Resting FBF and FVC were higher in males compared to females across all conditions (*p* = 0.024; *p* = 0.025, respectively). Blockade condition significantly altered FBF and FVC (*p* < 0.001 for both) but there was no effect of altitude (*p* = 0.330; *p* = 0.718, respectively). During exercise, ΔFBF was influenced by condition (*p* < 0.001), but not by sex (*p* = 0.696) or altitude (*p* = 0.813). Similarly, ΔFVC was different across conditions (control: 9.4 ± 2.3 mL/min/mmHg/FAV; β‐blockade: 11.4 ± 12.8 mL/min/mmHg/FAV; α‐β‐blockade: 3.9 ± 1.1 mL/min/mmHg/FAV; *p* < 0.001), with no effect of sex (*p* = 0.646) or altitude (*p* = 0.889). These results suggest males and females do not respond differently to exercise at altitude, and light‐intensity exercise hyperemia may be preserved during early acclimatization. α‐adrenergic receptors appear important for exercising blood flow, but β‐adrenergic receptors may not be critical in this response.

## 1 INTRODUCTION

During exercise, blood flow is increased to the active skeletal muscles to meet the elevated oxygen demand through changes in peripheral vascular tone and cardiac output. With the onset of exercise, feed‐forward mechanisms in higher motor centers of the brain signal the sympathetic nervous system to regulate the exercise response (i.e., central command) (Ishii et al., 2016). In addition, an array of feedback mechanisms including the exercise pressor reflex respond to changes in mechanical stretch and metabolite accumulation (McCloskey & Mitchell, 1972). Together, these mechanisms increase sympathetic activity to increase heart rate, stroke volume, and vascular tone, ultimately elevating cardiac output to meet the exercising blood flow demands.

Heightened sympathetic activity in the peripheral vasculature causes the release of neurotransmitters such as norepinephrine, binding to α‐adrenergic receptors and triggering vasoconstriction (Motiejunaite et al., 2021). Despite global sympathetic hyperactivity and vasoconstriction, the influence is lessened in exercising muscle vasculature via a phenomenon called functional sympatholysis. Sympatholysis occurs partly due to shear stress from increased blood flow through the arteries, triggering production of nitric oxide and other potent vasodilators and enabling exercise hyperemia (Jendzjowsky & Delorey, 2013b; Tschakovsky & Joyner, 2008). Differential distribution of α‐adrenergic receptors contributes to greater dilation of the distal vessels, enabling optimal perfusion to the active tissues (Folkow et al., 1971). Thus, regulation of the macrovasculature during exercise is a balance of sympathetic vasoconstriction and dilatory sympatholytic pathways.

Chronic hypoxia is a significant physiological stressor causing sympathoexcitation (Hansen & Sander, 2003). Despite millions of individuals living and traveling to high altitude (i.e., >2500 m) each year (Tremblay & Ainslie, 2021), thus exposing themselves to high altitude hypoxia, there is conflicting evidence on autonomic control of blood flow during hypoxic exercise. Hypoxia presents sympathetic hyperactivity and contradicting vasoconstriction and vasodilation due to reduced oxygen delivery, creating a unique environment for regulating exercising blood flow. In acute hypoxia, forearm blood flow is elevated during exercise (Casey et al., 2010, 2014; Dinenno, 2016; Wilkins et al., 2008); whereas evidence in chronic hypoxia suggests vascular resistance is augmented during acclimatization to altitude (Calbet et al., 2003; Hansen et al., 2022; Lundby et al., 2006, 2008; Tymko et al., 2020). However, current knowledge of high altitude vascular control relies on research in only male participants.

Evidence supports differences between males and females in their physiological responses to exercise (Casey et al., 2014; Hart et al., 2009; Just & DeLorey, 2017); specifically, females appear to have augmented blood flow and vasodilation in response to exercise compared to males. At rest, males show a relationship between total peripheral resistance and cardiac output with muscle sympathetic nerve activity, in which responses to sympathetic stressors are associated with vasoconstriction and elevated cardiac output (Charkoudian et al., 2005) that does not exist in females (Hart et al., 2009). Further, evidence suggests vasodilatory β‐adrenergic receptors are upregulated in young females (Ferrer et al., 1996; Kneale et al., 2000), contributing to greater dilatory signaling. Current evidence is contradictory as to whether males and females differ in their response to hypoxia (Casey et al., 2014; Jacob et al., 2021; Miller et al., 2019; Patel et al., 2014; Usselman et al., 2015), and very limited research in hypoxic exercise suggests females have greater changes in conductance (Casey et al., 2014), but does not elucidate underlying mechanisms. Jacob et al. (2025) found an important role of the β‐adrenergic receptors in females but not males in hypoxic vasodilation, highlighting a potential pathway for sex differences in the vascular response to hypoxic exercise. Therefore, we investigated differences between sexes in the blood flow response to hypoxic exercise and the contributions of adrenergic mechanisms. We hypothesized: (1) females would have an augmented vasodilatory response to handgrip exercise during hypoxia compared to males, which would be abolished during β‐adrenergic receptor blockade, and (2) both males and females would have similarly blunted blood flow responses to handgrip exercise under combined α‐ and β‐adrenergic blockade.

## 2 METHODS

### 2.1 Participants

Sixteen participants were recruited, but only 13 participants (8 M/5F; 27 ± 4 years, 176 ± 7 cm, 72.8 ± 10.9 kg) completed the full study due to dropout (*n* = 2) and inability to place a catheter (*n* = 1). This study was part of a multi‐institution collaborative expedition to the White Mountain Barcroft Station (California, USA, 3800 m) and all participants were members of the expedition. 2 females were taking oral contraceptives (tested during active phase), and 3 females had intra‐uterine devices. Due to the nature of the expedition, we were unable to control for menstrual cycle or contraceptive phase.

### 2.2 Protocol

Experiments were performed at low altitude (LA; Kelowna, BC, 340 m) and high altitude (HA; White Mountain, CA, 3800 m) during days 3–11 (5.9 ± 3.1 days) of acclimatization. Participants completed a Lake Louise Acute Mountain Sickness Questionnaire on the day of participation. The highest score recorded was a 5 (out of 12), which is classified as mild acute mountain sickness (Roach et al., 2018). No participant experienced other high altitude‐related sicknesses (high altitude pulmonary edema, high altitude cerebral edema) or was taking medications for altitude sickness or acclimatization (e.g. acetazolamide). Participants were tested once at each location, with an identical protocol, and abstained from caffeine, exercise, and alcohol for 12 h and food for 2 h prior to participation.

Baseline cardiovascular characteristics were measured, including heart rate (electrocardiogram lead II), brachial artery pressure (pressure transducer; Edwards VAMP System), oxygen saturation (SpO_2_; pulse oximetry, ADInstruments, ML320 Oximeter Pod, Australia), and brachial artery diameter and blood flow velocity (Doppler ultrasonography; Terason uSmart 330, 12 L probe), captured using custom data collection software (J. Lawley, University of Innsbruck). Brachial artery pressure was measured through a brachial artery catheter inserted by a medical doctor. Briefly, participants were given local anesthetic (Lidocaine) in their left forearm. The expedition physician inserted a catheter using ultrasound guidance, which was attached to a fluid column to continuously measure arterial pressure. In the same arm, the physician also inserted an antecubital venous catheter for blood sample collection. Following insertion, participants rested for 10–15 min prior to the initiation of the protocol.

Forearm blood flow (FBF) was calculated as FBF (mL/min/100 mL forearm volume [FAV]) = (V_mean_ × π × (diameter/2)^2^ × 60)/FAV. Forearm vascular conductance (FVC) was calculated as FVC (mL/min/mmHg/FAV) = (FBF/MAP) × 100. FAV was determined using water displacement, in which the amount of water displaced by submerging the forearm up to the elbow was weighed, with the weight of the hand subtracted to calculate FAV. Arterial and venous blood samples were collected to assess partial pressure of oxygen and carbon dioxide, oxygen saturation, hematocrit, hemoglobin, and sex hormones. Participants performed 3 maximal voluntary contractions (MVCs) for 5 seconds each on the blockade arm (brachial artery catheter), with a minimum 1‐min rest between each. The highest MVC value was then used to determine the target for the exercise bout (25% of MVC).

Participants performed 3 identical trials of rhythmic handgrip (RHG) of 3 min at 25% maximal voluntary contraction (MVC) at a duty cycle of 2:1 with their left hand. As this protocol was part of a wider study, a new 2‐min baseline was taken immediately prior to the start of RHG each time. Ultrasound was performed throughout, with the 2nd min of both baseline and RHG used for analysis. Arterial and venous blood samples were taken in the last minute of baseline and RHG. The first trial was conducted during an arterial saline drip only. The second trial occurred during an intra‐arterial infusion of propranolol, a β‐adrenergic receptor antagonist, via brachial artery catheter at a rate of 10 μg/100 mL FAV for 10 min (loading dose), then maintained for the remainder of the protocol at 5 μg/100 mL FAV (maintenance dose) (Richards et al., 2017). The third trial occurred during an intra‐arterial infusion of phentolamine, a non‐specific α‐adrenergic receptor antagonist, via brachial artery catheter at a rate of 10 μg/100 mL FAV for 10 min (loading dose), then maintained for the remainder of the protocol at 5 μg/100 mL FAV (maintenance dose) (Hansen et al., 2020). Phentolamine was infused in conjunction with continued infusion of propranolol, ensuring maintenance of the β‐blockade.

### 2.3 Data analysis

Baseline cardiovascular measures at LA and HA were averaged in the last minute of a 10‐min rest. Each bout of RHG had a baseline immediately preceding it; values were averaged over a 1‐min period prior to initiating exercise. Responses to RHG were averages on the 2nd minute, at which point participants had achieved steady state exercise. All statistical analyses were conducted using R (v 4.4.2; https://www.R‐project.org), lme4, lmerTest, and emmeans statistical packages. Demographics were compared with a two‐way (altitude × sex) repeated measures linear mixed‐effects model. A linear mixed‐effects model was used to test the relationships between the resting and delta physiological responses to exercise (FBF, FVC) across conditions. Delta responses were calculated as the 1‐min average from baseline subtracted from the 1‐min average from exercise. A model including sex and a second including sex and altitude as fixed effects were compared against a null model (i.e., one without sex and altitude as fixed factors) to assess if there are significant differences or interactions between condition, sex, and altitude on FBF and FVC. In all models, ID was considered a random effect, allowing for variable intercepts between participants. The fit of the model was assessed using Akaike's information criterion (AIC), and the model with the lowest AIC was used. However, we chose to include altitude and sex as fixed factors despite not improving the model because of the well‐established physiological effects of altitude on FBF and FVC and the central question pertaining to an effect of sex. When a significant *F* test was achieved, pairwise comparisons were made with a Tukey's post hoc analysis to account for multiple comparisons. All data are reported as mean ± standard deviation (SD).

## 3 RESULTS

**FIGURE 1:**
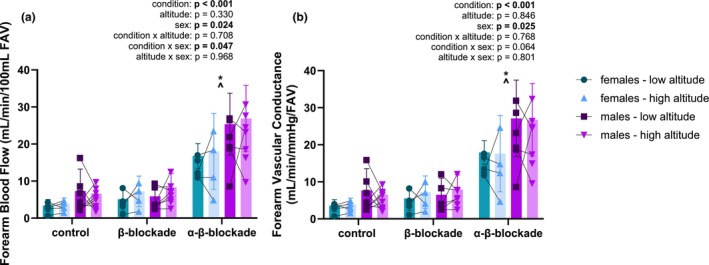
(a) Resting forearm blood flow across conditions and altitudes in females and males. Linear mixed‐effects model identified a main effect of blockade condition (*p* < 0.001) and sex (*p* = 0.024), but no effect of altitude (*p* = 0.330). The interaction between condition and sex was significant (*p* = 0.047). Post‐hoc analyses found no difference between control and β‐blockade (*p* = 0.668), but a significant difference between control and α‐β‐blockade (*p* < 0.001) and β‐blockade and α‐β‐blockade (*p* < 0.001). *n* = 5 for females, except in β‐blockade and α‐β‐blockade at high altitude (*n* = 4); *n* = 7 for males, except in control at high altitude (*n* = 8), and α‐β‐blockade at low altitude (*n* = 6); *p < 0.05 compared to control; ^*p* < 0.05 compared to β‐blockade. (b) Resting forearm vascular conductance across conditions and altitudes in females and males. There was a main effect of condition (*p* < 0.001) and sex (*p* = 0.025), but no effect of altitude (*p* = 0.846), as identified by linear mixed‐effects model. Interactions were not significant between any factors. Post‐hoc multiple comparisons identified a difference between control and α‐β‐blockade (*p* < 0.001) and between β‐blockade and α‐β‐blockade (*p* < 0.001), but no difference between control and β‐blockade (*p* = 0.757). *n* = 5 for females, except for β‐blockade and α‐β‐blockade at high altitude (*n* = 4); *n* = 8 for males, except during β‐blockade at low altitude and α‐β‐blockade at high altitude (*n* = 7) and α‐β‐blockade at low altitude (*n* = 6); *p < 0.05 compared to control; ^*p* < 0.05 compared to β‐blockade.

Altitude significantly increased resting HR (LA, 56.6 ± 4.5 vs. HA, 74.2 ± 5.5 bpm; *p* < 0.001) and MAP (LA, 91.1 ± 1.8 vs. HA, 97.3 ± 0.1 mmHg; *p* < 0.001), but did not change FBF (LA, 5.1 ± 2.2 vs. HA, 6.2 ± 1.5 mL/min/100 mL FAV; *p* = 0.294). There was no effect of sex on baseline HR, MAP, or FBF between altitudes (Table 1; control condition, Figure 1). Estrogen and progesterone were not different between males and females, but testosterone was significantly higher in males compared to females at LA (*p* = 0.005) and HA (*p* < 0.001; Table 1).

**TABLE 1:** Baseline cardiovascular and hematological characteristics at low and high altitude.

	Low altitude		High altitude		*p*‐values		
	Males	Females	Males	Females	Altitude	Sex	Interaction
HR (bpm)	53.4 ± 6.4	59.7 ± 6.1	70.3 ± 14.6	78.1 ± 17.9	**<0.001**	0.258	0.843
MAP (mmHg)	89.8 ± 7.4 (7)	92.4 ± 7.1	97.3 ± 5.6 (7)	97.4 ± 8.2	**<0.001**	0.730	0.526
SaO_2_ (%)	98.0 ± 0.9 (7)	97.6 ± 0.4	88.4 ± 3.3	88.7 ± 2.5	**<0.001**	0.956	0.694
pO_2_ (mmHg)	93.2 ± 9.2 (7)	94.6 ± 5.2	51.2 ± 6.6	54.5 ± 3.7	**<0.001**	0.493	0.645
pCO_2_ (mmHg)	38.5 ± 2.9 (7)	37.1 ± 3.5	27.9 ± 1.4	26.1 ± 1.3	**<0.001**	0.130	0.880
Hemoglobin (g/dL)	13.6 ± 0.8 (7)	12.6 ± 0.2	14.2 ± 0.7	12.7 ± 0.7	0.183	**<0.001**	0.392
Hematocrit (%)	41.6 ± 2.5 (7)	38.6 ± 0.8	43.5 ± 2.2	38.9 ± 2.1	0.205	**<0.001**	0.384
Estrogen (pmol/L)	91.8 ± 24.7 (5)	297.8 ± 201.6 (4)	95.4 ± 32.2	399.0 ± 415.6	0.263	0.065	0.276
Progesterone (nmol/L)	1.3 ± 0.4 (5)	8.6 ± 15.1 (4)	1.6 ± 0.2	6.6 ± 7.1	0.788	0.129	0.691
Testosterone (nmol/L)	12.0 ± 4.8 (5)	0.7 ± 0.3 (4)	13.8 ± 7.0	1.1 ± 0.4	0.327	**0.001**	0.476

*Note*: Males *n* = 8, females *n* = 5 unless otherwise specified in parentheses. A two‐way (altitude × sex) repeated‐measures linear mixed‐effects model was fitted with ID as a random effect for all demographics. Bold are provided except in cases where the value exceeds the threshold of <0.001.

### 3.1 Effects of α‐β‐adrenergic antagonists

Blockade condition significantly influenced FBF (*p* < 0.001), but there was no main effect of altitude (*p* = 0.330; Table 2). Individual comparison identified α‐β‐blockade significantly elevated FBF compared to control (*p* < 0.001) and β‐blockade (*p* < 0.001). FBF in control compared to β‐blockade was not different (*p* = 0.668). The day of acclimatization did not influence resting FBF (*p* = 0.842). Resting FBF was higher in males compared to females in all conditions (main effect of sex *p* = 0.024; Figure 1a). However, there was a significant interaction between condition and sex (*p* = 0.047), while individual interactions suggest resting FBF in males may be significantly higher during α‐β‐blockade from β‐blockade compared to females (*p* = 0.076), but no other interactions were significant. Post‐hoc analyses by condition found males had greater resting FBF compared to females during α‐β‐blockade (*p* = 0.001), but were not different during control (*p* = 0.159) or β‐blockade (*p* = 0.736).

**TABLE 2:** Forearm blood flow and vascular conductance during handgrip.

Low altitude			High altitude			*p*‐values		
Control	β‐Blockade	a‐β‐blockade	Control	β‐Blockade	a‐β‐blockade	Condition	Altitude	Sex
66.4 ± 10.3	65.8 ± 7.6	63.4 ± 9.2	84.4 ± 18.6	81.4 ± 14.1 (4)	80.3 ± 14.0 (4)	0.098	**<0.001**	0.105
57.7 ± 7.3	52.4 ± 8.1 (7)	52.9 ± 7.3 (7)	73.5 ± 15.5	68.0 ± 15.7	68.7 ± 17.3			
100 ± 8.3	98.2 ± 5.4 (4)	99.3 ± 7.8	102.6 ± 8.2	95.7 ± 4.4 (3)	100.2 ± 6.8 (3)	0.531	0.443	0.329
100.5 ± 10.3 (6)	101.9 ± 7.5 (6)	104.5 ± 9.5 (7)	99.5 ± 5.4	109.4 ± 10.3 (6)	106.6 ± 6.2 (7)			
15.0 ± 6.8	17.5 ± 8.6	21.6 ± 2.3	11.0 ± 5.8	17.3 ± 8.5 (3)	23.2 ± 8.0 (4)	**<0.001**	0.718	**0.026**
17.2 ± 4.7	18.8 ± 7.5 (7)	32.3 ± 7.3 (7)	17.6 ± 5.1	23.5 ± 7.9	32.3 ± 7.3 (7)			
15.0 ± 6.7	20.1 ± 7.0 (4)	21.8 ± 1.8	10.5 ± 5.0	15.1 ± 9.5 (2)	22.7 ± 9.4 (3)	**<0.001**	0.741	**0.039**
16.4 ± 4.7 (6)	16.3 ± 4.9 (6)	31.2 ± 7.5 (7)	17.8 ± 5.1	20.0 ± 5.4 (6)	30.5 ± 5.7 (7)			

*Note*: Males *n* = 8, females *n* = 5 unless otherwise specified in parentheses. A three‐way (condition × altitude × sex) repeated‐measures linear mixed‐effects model was fitted with ID as a random effect for all variables during exercise. Bold are provided except in cases where the value exceeds the threshold of <0.001.

There was a main effect of condition on resting FVC (*p* < 0.001), but no effect of altitude (*p* = 0.846; Figure 1b). Post‐hoc comparisons identified a difference in FVC between control and α‐β‐blockade (*p* < 0.001) and between β‐blockade and α‐β‐blockade (*p* < 0.001), but no difference between control and β‐blockade (*p* = 0.757). The day of altitude exposure did not influence resting FVC (*p* = 0.941). Comparing sexes, resting FVC was significantly higher in males across all conditions (*p* = 0.025), but a potential interaction between condition × sex (*p* = 0.064) may exist. Other interactions were not significant between condition × altitude (*p* = 0.768), altitude × sex (*p* = 0.801), and condition × altitude × sex (*p* = 0.942).

### 3.2 Effects of α‐β‐adrenergic antagonists on handgrip exercise

**FIGURE 2:**
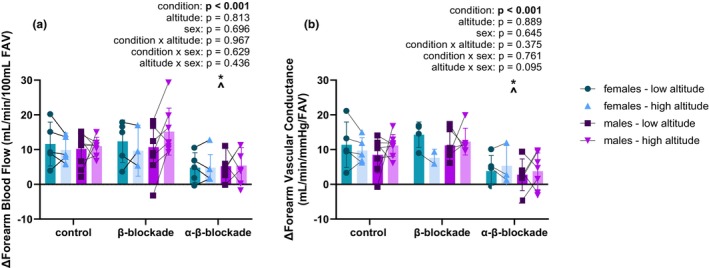
(a) The change in forearm blood flow across conditions and altitudes in females and males. There was a main effect of condition (*p* < 0.001), but no effect of altitude (*p* = 0.813). Interactions were not significant between any variables. Post‐hoc analyses identified a difference between α‐β‐blockade compared to control (*p* = 0.001) and β‐blockade (*p* < 0.001) conditions, but no difference between control and β‐blockade (*p* = 0.299). There was no effect of sex on the change in forearm blood flow (*p* = 0.696). *n* = 5 for females, except in β‐blockade at high (*n* = 3) altitude, and α‐β‐blockade at high altitude (*n* = 4); *n* = 8 for males, except β‐blockade at low altitude (*n* = 7) and α‐β‐blockade at low (*n* = 6) and high altitude (*n* = 7); *p < 0.05 compared to control; ^*p* < 0.05 compared to β‐blockade. (b) The change in forearm vascular conductance across conditions and altitudes in females and males. There was a main effect of condition (*p* < 0.001), but no main effect of altitude (*p* = 0.889). Interactions were not significant between any variables. Post‐hoc multiple comparisons found a difference between control and α‐β‐blockade (*p* < 0.001) and between β‐blockade and α‐β‐blockade (*p* < 0.001), but no difference between control and β‐blockade (*p* = 0.631). Sex did not significantly influence the change in forearm vascular conductance (*p* = 0.645). *n* = 5 for females, except for β‐blockade at low (*n* = 4) and high altitude (*n* = 2) and α‐β‐blockade at high altitude (*n* = 3); *n* = 6 for males, except control at high altitude (*n* = 8) and α‐β‐blockade at high altitude (*n* = 7); *p < 0.05 compared to control; ^*p* < 0.05 compared to β‐blockade.

The absolute FBF and FVC responses to RHG are reported in Table 2. In this section we will discuss the relative change (i.e., delta relative to baseline). Blockade condition exerted a main effect on the change in FBF from rest to RHG (*p* < 0.001; Figure 2a). Altitude exposure did not influence ΔFBF (*p* = 0.813), and day of acclimatization did not alter the change in FBF to RHG (*p* = 0.251). All interactions between condition, altitude, and sex were not significant (Figure 2). Multiple comparisons identified ΔFBF was reduced during α‐β‐blockade compared to control (*p* = 0.001) and β‐blockade (*p* < 0.001). There was no difference in the change in FBF between control and β‐blockade (*p* = 0.299). Sex did not influence ΔFBF (*p* = 0.696).

The change in FVC during RHG was significantly altered due to condition (*p* < 0.001), but altitude (*p* = 0.889; Figure 2b) did not have an effect. The interactions between condition and altitude (*p* = 0.375; Figure 2b), condition and sex (*p* = 0.761), and altitude and sex (*p* = 0.095) were not significant. Post‐hoc multiple comparisons found a difference between control and α‐β‐blockade (*p* < 0.001) and between β‐blockade and α‐β‐blockade (*p* < 0.001), but no difference between control and β‐blockade (*p* = 0.631). Day of acclimatization had a main effect on ΔFVC (*p* = 0.018), but the individual acclimatization day coefficient was not significant (*p* = 0.068), and there was no effect of acclimatization day on the FVC response to condition (*p* = 0.973). There was no effect of sex on ΔFVC (*p* = 0.645).

## 4 DISCUSSION

The aim of this study was to examine sex differences in the blood flow responses to hypoxic exercise and elucidate the direct contributions of the adrenergic receptors. The main findings are three‐fold: (1) the blood flow and conductance response to handgrip exercise was not different with altitude exposure; (2) there appears to be an effect of sex in resting blood flow and conductance, but contrary to our hypothesis, males had greater blood flow and conductance, with no sex differences in the response to exercise (3) α‐adrenergic receptors play a crucial role in regulating the blood flow response to exercise, but our exploratory data does not support a significant role for β‐adrenergic in this response. Together, these data suggest males and females may differ in regulation of resting blood flow, but do not respond differently to light exercise, regardless of hypoxia exposure. Potential interactions between adrenergic receptor contribution and altitude with sex may indicate a role of sex and sex‐specific hormones in regulation of resting blood flow.

### 4.1 Effects of altitude on exercise hyperemia

Contrary to our hypothesis, we showed no difference in the blood flow or conductance response to rhythmic handgrip exercise with altitude exposure. This contradicts other findings that suggest reduced skeletal muscle vasodilation and blood flow with altitude acclimatization (Calbet et al., 2003; Hansen et al., 2022; Lundby et al., 2008). It is important to note that due to the small sample size, the risk of a type 2 error is significant. Post‐hoc power analysis suggests a very low power to detect an effect of altitude on the conductance response (power = 0.10). Nonetheless, the forearm vascular response to exercise in acute hypoxia results in an increase in blood flow (Casey et al., 2010, 2014; Dinenno, 2016; Joyner & Casey, 2014; Wilkins et al., 2008); thus, there is a transition from compensatory to blunted hypoxic vasodilation with acclimatization. As we tested participants during early acclimatization (days 3–11), it is possible we captured this window of transition, explaining the absence of an altitude effect. Indeed, initial changes in blood volume, cardiac output, blood pressure, ventilation, and arterial oxygen content are restored within the first week or two of acclimatization (Bender et al., 1989; Naeije, 2010). As our participants demonstrated elevated blood pressure, it is likely these early processes of acclimatization were still occurring. The day of testing at altitude did significantly influence the change in forearm vascular conductance during exercise, suggesting that although altitude did not impact the overall results, some effects of acclimatization were observed in our results.

With acclimatization, there is significant plasma volume constriction (Beidleman et al., 2017; Roche et al., 2022) which enhances hematocrit and hemoglobin concentration and aids oxygen delivery. Chicco et al. (2018) suggest mitochondrial respiratory efficiency is improved within 2 weeks of acclimatization, reducing metabolic demand and enabling a blunted exercise blood flow response. In contrast to other acclimatization studies, we did not observe an increase in hemoglobin concentration or hematocrit; although likely due to a small sample size, it is possible the capacity for improved oxygen delivery may be reduced in our participants. Further, Hansen et al. (2022) found tonically increased α‐adrenergic signaling contributes to reduced hypoxic exercise hyperemia following 3 weeks of altitude exposure. Heightened α‐adrenergic activity results from chronically elevated sympathetic activity but is modulated by blunted neurovascular transduction at altitude (Berthelsen et al., 2020), which may occur more quickly (i.e., with acute hypoxia) (Shafer et al., 2023; Steele et al., 2021). Clearly, a multitude of contrasting mechanisms contribute to regulating exercise with chronic hypoxia exposure and could explain our similarities between low and high altitude. More research is needed to understand the effect of altitude acclimatization on exercising forearm blood flow.

### 4.2 Effect of sex on forearm blood flow and conductance

In contrast with previous findings of sex differences in resting conductance (Casey et al., 2014; Gentilin et al., 2022; Hansen et al., 2022; Tymko et al., 2020), we found higher conductance in males. Greater conductance previously reported in females is likely due to higher β‐adrenergic receptor densities and sensitivity in females compared to males (Al‐Gburi et al., 2017; Kneale et al., 2000), which are upregulated by estrogen (Ferrer et al., 1996). Crucially, we cannot rule out the possibility of a type 2 error in our data, due to the small sample size and missing data. For example, there was no significant difference in estrogen levels between males and females (*p* = 0.065; Table 1), likely due to variability in menstrual cycle phase and the use of some form of contraceptive by all females. There is conflicting information on the influence of menstrual cycle phase and contraceptive use on exercise hyperemia (Cottingham et al., 2001; Gonzales et al., 2007; Hunter et al., 2006; Limberg et al., 2010; Lynn & McCord, 2007; Pereira & Edgell, 2024); thus, differences in contraceptive use, cycle phases, and a small sample size of females may have contributed to the lack of sex differences.

Our results suggest no difference in the exercising hyperemic responses in males and females to both normoxic and hypoxic mild‐intensity exercise. This contradicts a large body of literature supporting greater conductance responses in females in normoxia (Gonzales et al., 2007; Kneale et al., 2000; Parker et al., 2007) and hypoxia (Casey et al., 2014; Jacob et al., 2021). One critical difference between our study and the previous literature is the exercise intensity; we employed a mild‐intensity stimulus, while others have performed exercise to exhaustion (Gonzales et al., 2007; Parker et al., 2007). It is possible that a higher intensity stimulus is needed to elicit sex differences in the reactive hyperemia response. However, Limberg et al. (2010) showed sex differences in lower but not higher intensities, and Casey et al. (2014) found differences in hypoxic exercise at 10% and 20% forearm exercise.

### 4.3 Adrenergic receptor contributions to hypoxic exercise

Importantly, our study is the first we are aware of to include female participants when examining adrenergic contribution to functional sympatholysis at altitude. We showed a significant contribution of the adrenergic receptors to both resting and exercising conductance. Contrary to our hypothesis, however, β‐adrenergic receptor blockade did not alter exercise hyperemia in males or females. Other studies have found an important role of β‐adrenergic receptors in modulating blood flow in response to sympathetic stressors (Eklund & Kaijser, 1976; Kneale et al., 2000; Samora et al., 2019; Silva & Zanesco, 2010), but Cooper et al. (2021) did not find a significant role of β‐adrenergic receptors in the response to external muscle stimulation in rats. These results may suggest β‐adrenergic receptors respond differently in the exercising versus non‐exercising vasculature. Further, we did not observe a difference between males and females, thus not supporting a difference in adrenergic control during exercise between sexes. In this classic study, Kneale et al. (2000) found greater vasodilatory responses to β‐adrenergic agonists and blunted constrictor responses to norepinephrine. We observed a main effect of sex in resting blood flow and conductance, with an interaction between sex and condition on blood flow, and approaching significance in conductance (*p* = 0.064). In line with Kneale et al. (2000), this larger increase in resting blood flow under combined α‐β‐adrenergic blockade suggests males may have heightened α‐adrenergic receptor sensitivity and/or greater sympathetic restraint (Figure 1). However, in the study by Kneale et al. (2000), β‐adrenergic blockade eliminated differences between males and females in the response to norepinephrine, indicating sex differences are due to β‐adrenergic receptors. In contrast, we observed reduced α‐adrenergic sensitivity in females, suggesting sex differences in α‐adrenergic receptors. Thus, a difference in contributions of α‐ and β‐adrenergic receptors between males and females remains unclear.

In support of our hypothesis, exercise hyperemia was severely blunted during combined α‐β‐adrenergic blockade. This is in agreement with a large body of literature, illustrating the role of the adrenergic receptors in regulation of vascular tone (Casey et al., 2014; Dinenno et al., 2002; Eklund & Kaijser, 1976; Hansen et al., 2020, 2022; Limberg et al., 2010). Interestingly, we found combined α‐β‐adrenergic receptor blockade severely blunts exercise hyperemia regardless of altitude. This may be explained by a higher resting flow that already meets the increased metabolic demand seen with exercise, or could also be explained by a ‘ceiling effect’ of functional sympatholysis. Under combined α‐β‐adrenergic blockade, resting blood flow was so significantly elevated that a further increase with exercise was not possible. Although unable to confirm the presence of a true ‘ceiling’ with a high flow control, it is clear the adrenergic receptors are crucial for exercise hyperemia. However, more work is needed to elucidate the effects of sex and altitude acclimatization on adrenergic contribution to functional sympatholysis.

It is important to note there are a multitude of other mechanisms that contribute to the vascular response to exercise, which likely explain the increase in conductance under combined α‐β‐adrenergic receptor blockade. Although not the focus of this study, nonadrenergic mechanisms such as neuropeptide Y and ATP contribute to sympathetic vasoconstriction during exercise (Buckwalter et al., 2003, 2004). Nitric oxide is an important regulator of functional sympatholysis, triggered by shear stress and other chemical stimuli released during exercise to moderate the degree of vasoconstriction (Nosarev et al., 2014; Silva & Zanesco, 2010). Just and DeLorey (2017) suggest nitric oxide‐mediated sympatholysis is modulated by sex; indeed, evidence suggests nitric oxide is more important for sympatholysis in females than males (Dinenno & Joyner, 2003; Jendzjowsky & DeLorey, 2013a). Although we did not observe any sex‐related differences in the exercising conductance response, it is possible there are differing contributions of these mechanisms that result in a similar outcome. Likely, there are redundant mechanisms contributing to the blunting of sympathetic vasoconstriction during exercise. Others support interactions between various mechanisms, such as nitric oxide with prostaglandins (Boushel et al., 2002), endothelial‐derived hyperpolarizing factor (Bauersachs et al., 1996; Schildmeyer & Robert M. Bryan, 2002), and adenosine and potassium (Casey et al., 2013; Dua et al., 2009).

### 4.4 Methodological considerations

As is the nature of all expedition and field research, we were unable to control for all variables. While at altitude, two participants tested positive for COVID‐19 soon after participation. It is possible they had already contracted it when they participated in our study, or that other participants had COVID‐19 but tested negative. Due to the large list of symptoms and effects of COVID‐19, it is possible that the physiological responses to altitude and exercise would be altered. However, we did not identify either of these participants as outliers, so their data was included in the results. Further, due to drop‐out and a short window for testing on the expedition, we are limited in the number of participants. Specifically, in one condition (forearm vascular conductance response in females during α‐β‐blockade at high altitude) there are only 2 participants; therefore, we cannot draw any conclusions specific to this condition. A larger cohort could clarify some of our findings. Post‐hoc simulation‐based power analyses using the fitted mixed‐effects model (condition, altitude, and sex) of the change in forearm vascular conductance indicate increasing sample size up to 300 participants does not improve statistical power to detect an effect of sex. This suggests the lack of statistical significance in our findings is not solely attributable to a small sample size.

Given the time constraints of expedition research, we were unable to control for menstrual phase or contraceptive use. As reflected in circulating hormone concentrations (Table 1), this resulted in large variability in estrogen and progesterone levels within female participants. It is well known that circulating catecholamines change with altitude exposure and acclimatization and respond differently to exercise with altitude (Mazzeo et al., 1991, 1994, 1995, 1998). Differences in catecholamine concentrations between low and high altitude tests may influence our results.

## 5 CONCLUSION

Our findings show a complex, multifactorial picture of the control of exercising vasculature between males and females at altitude. Altitude exposure did not influence blood flow or vascular conductance; thus, it is possible the blood flow response to light‐intensity exercise is preserved at altitude. We found differences between males and females in resting vascular conductance; however, males had greater resting conductance, contrary to our hypothesis. We found no effect of sex on the vascular response to exercise, in contrast to a large body of literature suggesting otherwise. Finally, combined α‐β‐adrenergic receptor blockade significantly blunted the blood flow response to exercise similarly between low and high altitude. This highlights the importance of α‐adrenergic receptors in functional sympatholysis regardless of altitude exposure. However, β‐blockade showed no difference in blood flow or vascular conductance during exercise, suggesting β‐adrenergic receptors are not necessary for forearm exercise hyperemia. There is extremely limited research in female physiology at high altitude. Despite evidence that females regulate their vasculature differently (Casey et al., 2014; Charkoudian et al., 2005; Hart et al., 2009, 2011), our current understanding of the influence of hypoxia and altitude on vascular reactivity comes largely from investigations in males. Further work exploring sex differences in the exercise response in hypoxia is needed.

## AUTHOR CONTRIBUTIONS

This study took place at the University of British Columbia – Okanagan and during a 2‐week sojourn to the White Mountain Barcroft Research Station in California. L.E.M., study design, collection and assembly of data, data analysis and interpretation, manuscript preparation. E.R.V., study design, collection and assembly of data, manuscript preparation. L.S., study design, collection and assembly of data, manuscript preparation. M.E., collection and assembly of data, manuscript preparation; K.F., collection and assembly of data. J.B., collection and assembly of data. C.G., collection and assembly of data. D.M., collection and assembly of data. S.v.D., study design, collection and assembly of data. J.A., collection and assembly of data, financial support, provision of study facilities. J.L., collection and assembly of data, data analysis and interpretation. P.N.A., provision of study facilities, manuscript preparation. T.G., collection and assembly of data, manuscript preparation. M.S., collection and assembly of data, data analysis and interpretation, manuscript preparation. J.M., study design, collection and assembly of data, manuscript preparation. C.D.S., study design, collection and assembly of data, data analysis and interpretation, manuscript preparation, financial support.

## FUNDING INFORMATION

L.E.M. was supported by an NSERC Canadian Graduate Research Scholarship – Master's. C.D.S. is supported by NSERC (RGPIN 06637) and a HSFC Joint National and Alberta New Investigator Award (HSFC NNIA Steinback).

## CONFLICT OF INTEREST STATEMENT

None to declare.

## ETHICS STATEMENT

This study was approved by the University of Alberta Health Research Ethics Board (Pro00096808), the University of British Columbia (H22‐01091), and Health Canada (NOL264793).

## CONSENT

Written informed consent was obtained from all participants prior to participation, and the study followed the declaration of Helsinki.

## Data Availability

All supplementary data can be provided by contacting the corresponding author.
